# Development of a feeding simulation to evaluate how feeding distribution in aquaculture affects individual differences in growth based on the fish schooling behavioral model

**DOI:** 10.1371/journal.pone.0280017

**Published:** 2023-02-02

**Authors:** Yuki Takahashi, Kazuyoshi Komeyama

**Affiliations:** Faculty of Fisheries Sciences, Hokkaido University, Hakodate, Hokkaido, Japan; National Sun Yat-sen University, TAIWAN

## Abstract

In this study, we developed a feeding simulation using the fish schooling behavior model to evaluate growth differences from the feeding spatial distribution. In the proposed simulation, feeding behavior was modeled using the fish schooling model to simulate the amount of feed consumed by each individual. Next, body mass growth was calculated based on the amount of feed consumed. A 3.0-m diameter aquaculture tank was used for the simulation. We used three feeding methods to evaluate how feeding distribution affected growth: Feeding A, B, and C. The feed was distributed in a square pattern with one side length of 1.5, 1.0, or 0.5 m for Feeding A, B, and C groups, respectively. The results revealed that individual differences in body mass resulting from each feeding method differed greatly. The individual difference was largest in the Feeding C group. Here, maximum swimming speed was assumed to be proportional to total length. The feeding area of Feeding C was narrow; therefore, the first individual to arrive in the feeding area dominated the feed. Large individuals accessed the feed more easily than did small individuals. Consequently, the growth of large individuals became more rapid, and the individual differences became large in Feeding C. A rearing test can be conducted in a short time, and the optimal aquaculture operation was easily determined using the proposed simulation method. We concluded that the proposed simulation is useful as a decision-making tool for aquaculture management.

## Introduction

The demand for fish has grown dramatically in recent years. Fisheries production from aquaculture has been increasing and exceeded production from fishing vessels in 2015 [1]. Feed is an important aquaculture management component. Less feed leads to less growth of reared fish, whereas overfeeding leads to feed waste. Therefore, a moderate feeding amount is required to minimize economic loss. The FAO reported that feeding cost is the main cost component on aquaculture farms, accounting for 60% of aquaculture production costs [2]. The relationship between the feeding amount and the growth of reared fish has been reported in many studies to determine the optimal amount to feed [3]. Another study reported that the competition for feed is an important factor when rearing fish over a long period [4]. An individual with high competitive ability preferentially accesses food resources; therefore, a highly competitive individual has a high growth rate, and vice versa. Consequently, the competition for feed leads to individual differences in growth. Individual differences in growth increase variability and instability therein.

Rearing experiments can be conducted to determine the optimum feeding operation. For example, Handeland *et al*. [5] performed a rearing experiment targeting Atlantic salmon, *Salmo salar*, at different water temperatures to determine the water temperature that maximizes feed conversion efficiency, *FCE*, defined as the ratio of feed intake to body mass. Similar studies have been widely conducted [6]. Other studies have focused on the spatial distribution of feeding. For example, Jørgensen *et al*. [7] conducted a rearing test in tanks with standing and flowing water. The feed was distributed evenly by water flow in the tanks with flowing water, whereas the feed clumped in the tanks with standing water. The authors concluded that the feed intake variability was small in the tank with flowing water. Therefore, the feeding method, such as the amount and its distribution, affected the final growth of fish.

We hypothesized that the growth difference from feeding in a spatial distribution was determined by the spatial behavior of individual fish in the tank. To understand the growth difference caused by feeding with a particular spatial distribution, the growth history of each individual should be quantitatively evaluated. However, growth is generally evaluated by the mean body mass of reared fish, and growth of an individual fish is difficult to track. Furthermore, it is costly and time-consuming to conduct an actual rearing experiment, and a comparison of various feeding methods is difficult.

Simulation studies have been widely performed on the growth of reared fish to determine feeding efficiency without performing an actual rearing experiment. In some studies, the dynamic energy budget (DEB) theory [8] has been employed to simulate fish growth. For example, Alver *et al*. [9] applied DEB theory to larval stage cod, *Gadus morhua*. Føre *et al*. [10–12] conducted a series of simulation studies and developed a simulation model that included the fish behavioral model and DEB theory; they reported the simulated mean growth in body mass, but did not discuss the relationship between individual behavior and individual growth.

In this study, we developed a feeding simulation method based on the fish schooling model to clarify the relationship between individual fish behavior and growth. First, we describe our proposed simulation method and the feeding spatial distribution results. Next, we focus on the spatial distribution of the feed, which reportedly affects the growth of fish. Fish growth was simulated under several feeding spatial distribution conditions using the proposed simulation method. We hypothesized that growth differences according to the feeding spatial distribution were determined by the behavior of individual fish. Finally, we discuss the utility of the proposed simulation method for aquaculture farm management, including the feeding strategy.

## Material and methods

### Schematic of the proposed simulation model

**Fig 1:**
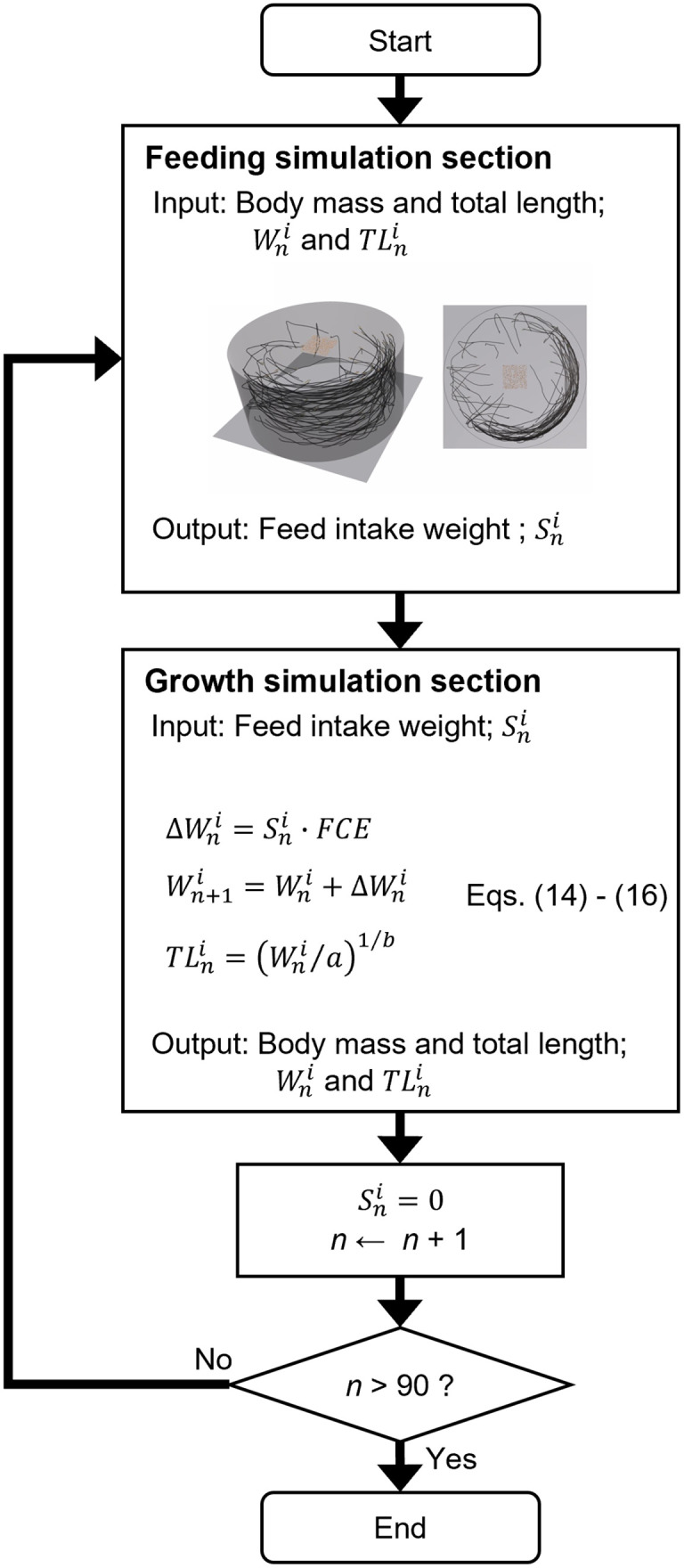
Schematic of the proposed simulation method. The proposed simulation was composed of a “Feeding simulation section” and a “Growth simulation section”. The simulation was repeated until the final day of the experiment (day 90). The details are shown in a flow chart in Fig 2.

Rainbow trout, *Oncorhynchus mykiss*, was targeted in this study. Fig 1 presents a schematic of the proposed simulation model. The proposed simulation was composed of a “feeding simulation” and a “growth simulation” section.

**Fig 2:**
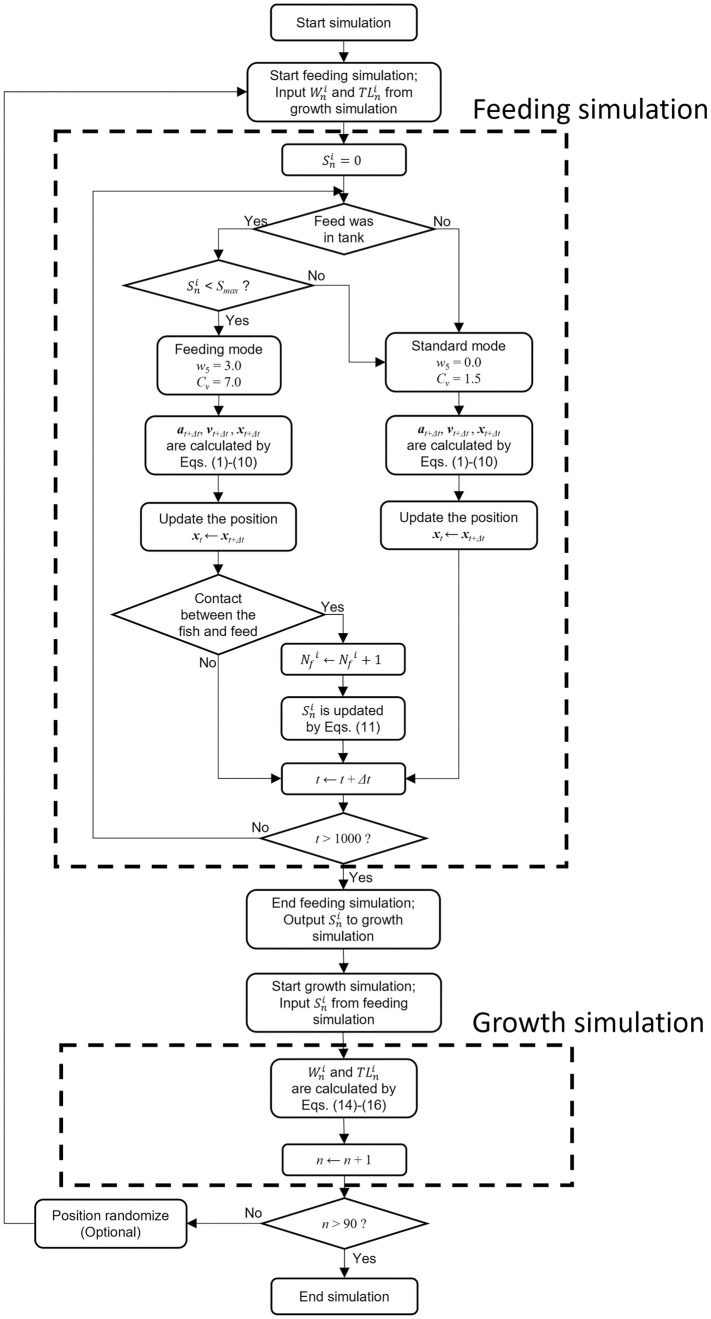
Flow chart of the simulation. For the feeding simulation, the input variables in the growth simulation section were $W_{n}^{i}$ and $TL_{n}^{i}$. The feeding simulation was conducted for 1,000 s. Finally, $S_{n}^{i}$ was calculated and exported to the growth simulation. The calculated $S_{n}^{i}$ was input into the growth simulation, which calculated $W_{n}^{i}$ and $TL_{n}^{i}$ and returned the values to the feeding simulation. This was repeated until the final day of the experiment (day 90). To confirm the spatial interaction between the fish and feed, a simulation in which randomization was performed every day after the feeding simulation was performed (see “Entire simulation set-up”).

Fig 2 contains a detailed flow chart of the proposed simulation model. The feeding simulation section was developed using the individual-based fish behavioral model. In this section, the weight of feed consumed by each individual, $S_{n}^{i}$, was the output. Here, $S_{n}^{i}$ is the weight of feed consumed by the *i*-th individual on the *n*-th day. The simulated feed intake weight, $S_{n}^{i}$, was transferred to the growth simulation section. In the growth simulation section, body mass, $W_{n}^{i}$, and total length, $TL_{n}^{i}$, were calculated based on the weight of feed consumed, $S_{n}^{i}$. The body mass and total length of each individual was transferred to the feeding simulation section. This simulation was repeated until the final day. Unity software 2018.3.12.f1 was used for the simulation and visualization.

### Feeding simulation

#### Schematic of the feeding simulation

Fish behavior, including movement against the feed, was simulated in the feeding simulation section. The weight of the feed consumed was output from the fish behavioral model.

Fish schooling behavior is determined by interference from other individuals. Several models have been developed to evaluate fish school movements from individual behavior, such as the boid model [13], the Sannomiya model [14, 15], and the stochastic model [16]. In this study, we used the boid model for the feeding simulation. The boid model was originally proposed to simulate bird flocking behavior [13], and has been applied to fisheries [17].

Here, the motion of an individual fish is expressed using Newton’s equation of motion as follows:

$$\begin{array}{l} W_{n}^{i}a_{t+\Delta t}=F_{t}\\ v_{t+\Delta t}=v_{t}+a_{t+\Delta t}\cdot \Delta t\\ x_{t+\Delta t}=x_{t}+v_{t+\Delta t}\cdot \Delta t \end{array}$$ (1)

where $W_{n}^{i}$ is fish body mass and ***a***_*t*_, ***v***_*t*_, ***x***_*t*_, and ***F***_*t*_ are the acceleration, velocity, position, and swimming force vectors at time, *t*, respectively. *Δt* is the time step of the simulation and is set to 0.025 s.

**Fig 3:**
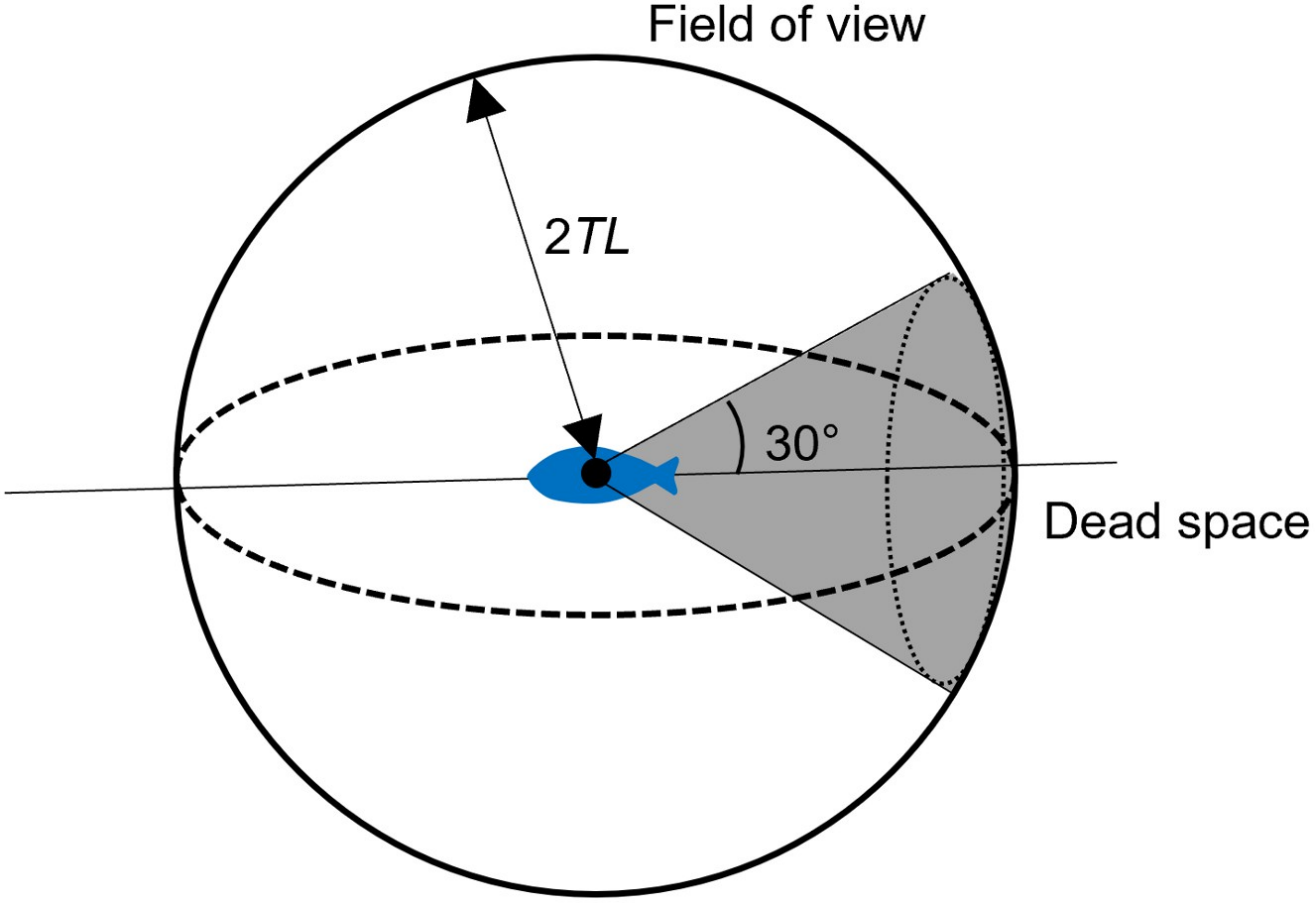
Schematic of an individual fish and its field of view. The field of view was defined to be a simple sphere with a radius twice the total length, $2TL_{n}^{i}$. The space behind within an angle of 30º was considered “dead space”.

***F***_*t*_ in Eq (1) is determined based on the boid model [13], in which schooling behavior is determined by an individual’s movement affected by neighboring individuals. Fig 3 presents an overview schematic of a modeled individual fish. The individual ***F***_*t*_ is determined by the other individuals within the field of view. Here, the field of view is assumed to be a simple sphere with a radius of 2*TL*, except that behind within an angle of 30° [17, 18]. We assumed that an individual can detect the feed regardless of the field of view.

**Fig 4:**
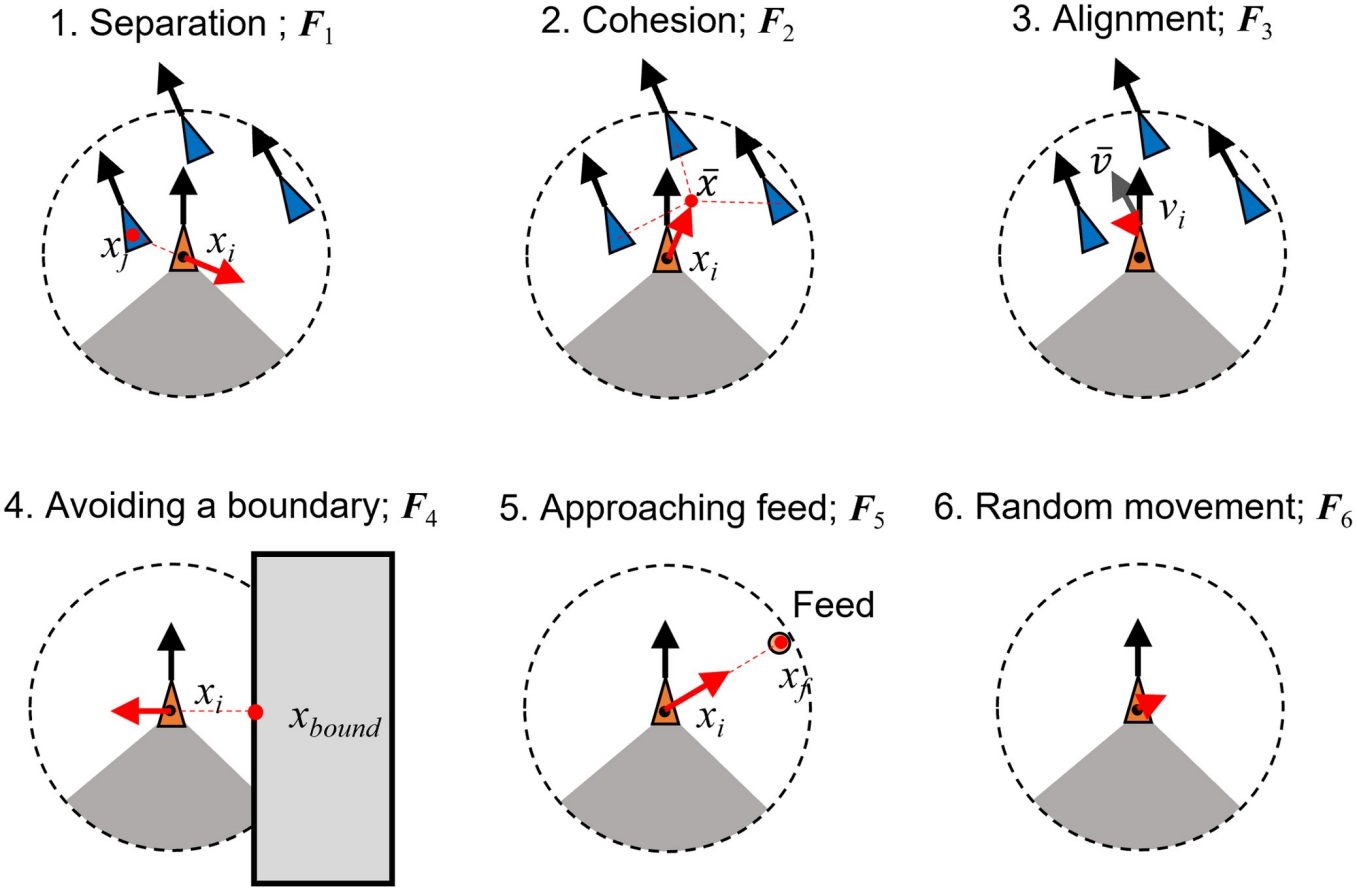
Overview of each force vector. Here, the behavioral model was based on the boid model [13]: 1. Separation, 2. Cohesion and 3. Alignment. Three additional rules were considered, 4. Avoiding a boundary, 5. Approaching feed, and 6. Random movement. Swimming force was expressed as the sum of the forces above.

Fig 4 provides an overview of swimming force. Here, fish schooling behavior is expressed by six rules: 1. Separation, ***F***_1_; 2. Cohesion, ***F***_2_; 3. Alignment, ***F***_3_; 4. Avoiding a boundary, ***F***_4_; 5. Approaching feed, ***F***_5_; and 6. Random movement, ***F***_6_. As a result, swimming force is calculated using the following equation:

$$F_{t}=\overset{6}{\underset{n=1}{\sum }}F_{n}$$ (2)

where ***F***_*n*_ is the *n*-th force vector. The details of each force are explained as follows.

#### Separation

The separation force vector, ***F***_1_, is a repulsive force from the nearest individual, described by the following equation:

$$F_{1}=w_{1}\frac{x_{i}-x_{j}}{\left|x_{i}-x_{j}\right|}$$ (3)

where ***x***_*i*_ is the position vector of the individual being considered, ***x***_*j*_ is the position vector of the nearest individual, and *w*_1_ is the weight coefficient of ***F***_1_.

#### Cohesion

The cohesion force vector, ***F***_2_, is defined as the vector that moves toward the center of other individuals in the field of view. As a result, the cohesion force vector is described using the following equation:

$$F_{2}=w_{2}\frac{\bar{x}-x_{i}}{\left|\bar{x}-x_{i}\right|}$$ (4)

where ***x***_*i*_ is the position vector of the individual being considered, and *w*_2_ is the weight coefficient of ***F***_2_. $\bar{x}$ is the center of gravity position vector of other individuals in the field of view, calculated using the following equation:

$$\bar{x}=\frac{\sum_{k=1}^{m}\;x_{k}}{m}$$ (5)

where ***x***_*k*_ is the position vector of the *k*-th individual in the field of view and *m* is the number of other individuals in the field of view.

#### Alignment

The alignment force vector, ***F***_3_, is the vector that moves in the same direction as other individuals, and is expressed using the following equation:

$$F_{3}=w_{3}\frac{\bar{v}-v_{i}}{\left|\bar{v}-v_{i}\right|}$$ (6)

where ***v***_*i*_ is the velocity vector of the individual being considered, and *w*_3_ is the weight coefficient of ***F***_3_. $\bar{v}$ is the average velocity vector of other individuals in the field of view, calculated using the following equation:

$$\bar{v}=\frac{\sum_{k=1}^{m}\;v_{k}}{m}$$ (7)

where ***v***_*k*_ is the velocity vector of the *k*-th individual in the field of view, and *m* is the number of other individuals in the field of view.

#### Avoiding a boundary

The avoiding boundary vector, ***F***_4_, is the repulsive force from the boundary, including a wall or bottom of the tank and water surface, and is expressed as follows.

$$F_{4}=w_{4}\frac{x_{i}-x_{bound}}{\left|x_{i}-x_{bound}\right|}$$ (8)

where ***x***_*i*_ is the position vector of the individual being considered, ***x***_*bound*_ is the position vector of the nearest point of the boundary in the field of view, and *w*_4_ is the weight coefficient of the avoiding force from the boundary.

#### Approaching feed

The approaching feed vector, ***F***_5_, is the attractive force to the feed. The force direction vector is expressed as follows:

$$F_{5}=w_{5}\frac{x_{f}-x_{i}}{\left|x_{f}-x_{i}\right|}$$ (9)

where ***x***_*i*_ is the position vector of the individual being considered, ***x***_*f*_ is the position vector of the feed, and *w*_5_ is the weight coefficient of the feeding behavior force vector. In this simulation, we assumed that the individual can detect the feed regardless of the field of view.

#### Random movement

The force vector of random movement, ***F***_6_, is considered based on uniformly distributed random numbers as follows:

$$F_{6}=\frac{w_{6}}{\sqrt{x_{1}^{2}+x_{2}^{2}+x_{3}^{2}}}{\left(x_{1},\;x_{2},x_{3}\right)}^{T}$$ (10)

where *w*_6_ is the weight coefficient of the random movement force, and *x*_1_, *x*_2_, and *x*_3_ are random numbers uniformly distributed over the range [–1, 1].

### Definition of feed intake during the feeding simulation

In this simulation, contact between the fish model and the feed model was defined as feed intake. The number of feed pellets contacted by each individual, $N_{f}^{i}$, was counted during the feeding simulation. The contacted feed was deleted immediately during the simulation. As a result, feed intake weight $S_{n}^{i}$ was calculated after the feeding simulation using the equation:

$$S_{n}^{i}=N_{f}^{i}\cdot W_{f}$$ (11)

where $S_{n}^{i}$ is the feed intake weight of the *i*-th individual on the *n*-th day and *W*_*f*_ is the weight of a grain of feed. The feed was assumed to be a simple sphere with diameter of 5.0 mm and a volume of 0.065 cm^3^. The density of the feed was the same as water; therefore, weight, *W*_*f*_, was 0.065 g. The sinking speed of the feed was set to 0.025 m/s.

### Parameters for the feeding simulation

In the simulation, fish behavior was divided into the “standard mode” and the “feeding mode”. Table 1 presents a schematic of the two swimming modes. If the feed was in the tank and the feed intake weight $S_{n}^{i}$ was smaller than the maximum feed intake weight *S*_*max*_, the individual was in “feeding mode”. Otherwise, the individual was in “standard mode”.

**Table 1:** Matrix of the two swimming modes.

		Feed in the tank	
		Yes	No
Appetite condition	*S*_*max*_ > *S*_*n*_^*i*^	Feeding mode	Standard mode
	*S*_*max*_ ≦ *S*_*n*_^*i*^	Standard mode	Standard mode

Some studies have investigated feed intake per day; however, we found no study focusing on maximum feed intake weight. Morales *et al*. [19] reported that the feed intake weight per day was 1.43–2.26% of fish body mass. In this study, we classified excess feeding based on the literature [20, 21]; the maximum feed intake, *S*_*max*_ was 4% of the body mass ($S_{max}=0.04W_{n}^{i}$).

The strength of each force was determined by the weight coefficients, *w*_*n*_ described as above. Table 2 lists the *w*_*n*_ values. The weight coefficients except for the approaching feed, *w*_5_, were fixed. However, *w*_5_ was determined by the mode of the individual. Here, *w*_5_ was set to 3.0 for the “feeding mode” and 0.0 for the “standard mode”.

**Table 2:** Weight coefficient, *w*_*n*_, of each force vector and coefficient of maximum velocity for each swimming mode.

	Standard mode	Feeding mode
*w*_1_	0.6	0.6
*w*_2_	0.4	0.4
*w*_3_	0.4	0.4
*w*_4_	1.0	1.0
*w*_5_	0.0	3.0
*w*_6_	0.2	0.2
*C*_*v*_	1.5	7.0

Swimming force was calculated using Eq (2), and acceleration, velocity, and position were determined using Eq (1). In this simulation, the maximum swimming velocity magnitude |***v***|_*max*_ was defined to consider the limit of fish swimming ability. If the velocity magnitude in Eq (1), |***v***|, exceeded the maximum velocity magnitude, the swimming velocity vector was corrected as follows:

$$v={\left|v\right|}_{max}\cdot e_{v}$$ (12)

where ***e***_*v*_ is the unit vector of the swimming velocity vector ***v***. Maximum swimming velocity, |***v***|_*max*_ was proportional to total length, *TL*, as follows:

$${\left|v\right|}_{max}=C_{v}\cdot TL_{n}^{i}$$ (13)

where *C*_*v*_ is the coefficient of the maximum velocity magnitude; Table 2 lists the values. The *C*_*v*_ value was modified by the swimming modes: *C*_*v*_ = 1.5 for the “standard mode” [22] and *C*_*v*_ = 7.0 for the “feeding mode” [23].

### Growth simulation

The amount of growth of each individual was determined by feed intake, $S_{n}^{i}$, based on the feeding simulation. We calculated the amount of body mass growth from the feed conversion efficiency, *FCE* = body mass gain/weight of feed intake, as follows:

$$\Delta W_{n}^{i}=S_{n}^{i}\cdot FCE$$ (14)

$$W_{n+1}^{i}=W_{n}^{i}+\Delta W_{n}^{i}$$ (15)

where *W* is the body mass, *ΔW* is the daily body mass gain, and *S* is the weight of the feed consumed. The subscripts *i* and *n* indicate the individual number and the rearing day, respectively. *FCE* is feed conversion efficiency; here, we assumed *FCE* to be 1.0 [19].

After the body mass was updated, total length $TL_{n}^{i}$ was calculated from the following allometric equation:

$$W_{n}^{i}=aTL_{n}^{i}{}^{b}\;\Leftrightarrow \;TL_{n}^{i}={\left(\frac{W_{n}^{i}}{a}\right)}^{\frac{1}{b}}$$ (16)

where $TL_{n}^{i}$ is the total length of the *i*-th individual on the *n*-th day and *a* and *b* are coefficients of allometry; here, *a* was 0.0209 and *b* was 2.843, respectively [24].

### Entire simulation set-up

**Fig 5:**
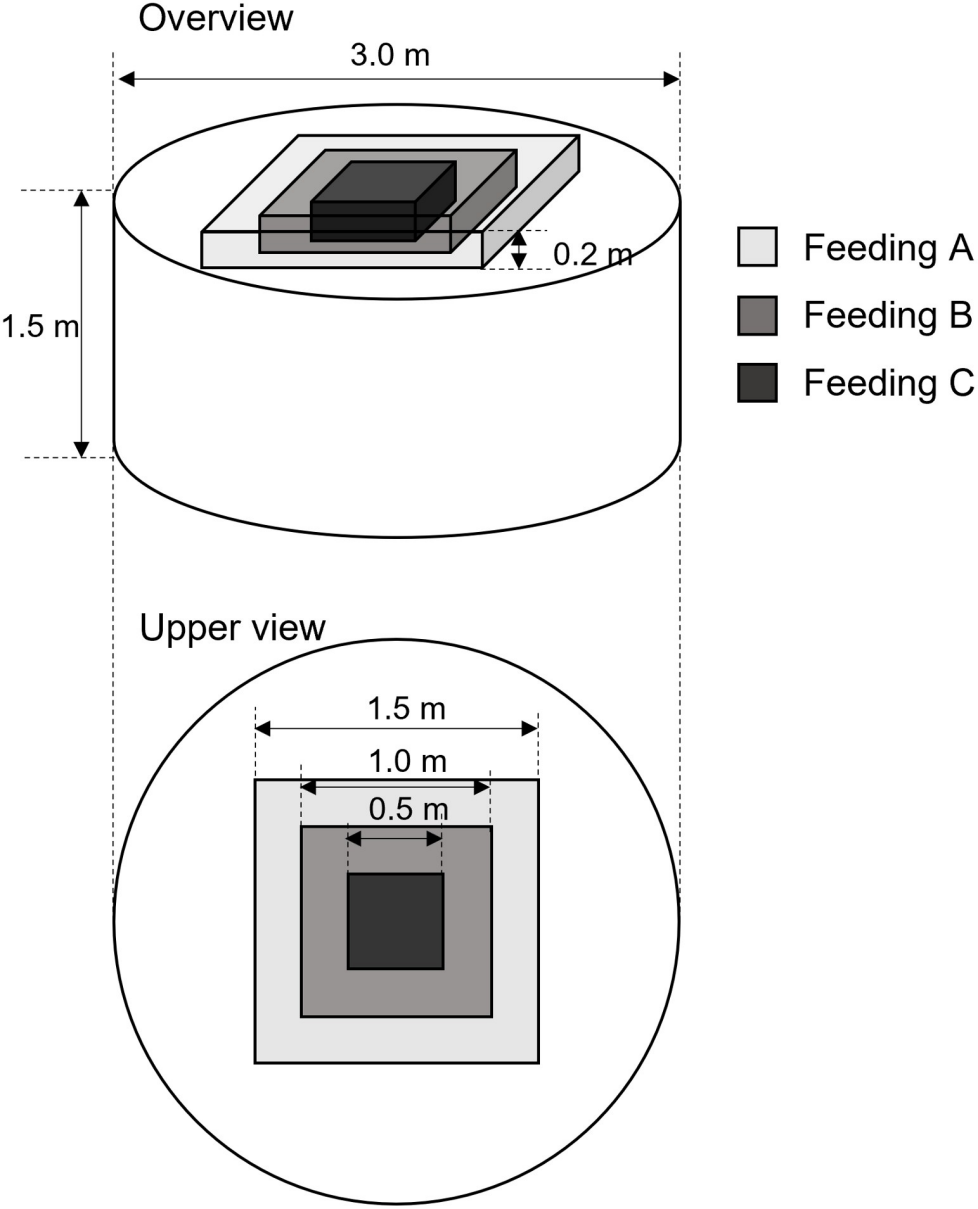
Simulation field. A 3.0-m diameter aquaculture tank with depth of 1.5 m was used with three feeding distributions in a square pattern: one side length was set to 1.5 m for the Feeding A, 1.0 m for the Feeding B, and 0.5 m for the Feeding C groups.

A 3.0-m diameter aquaculture tank with a depth of 1.5 m was used for the simulation (Fig 5). We simulated the three feeding methods named Feeding A, B, and C to evaluate the impact of the feeding method on growth, particularly the feed distribution (Fig 5). Feed was distributed in a square pattern for all methods, but the one-side length was different. The lengths were 1.5, 1.0, and 0.5 m for Feeding A, B, and C groups, respectively. The feed was homogeneously and randomly distributed using the “Random” function of Unity.

In total, 100 individuals were simulated in each feeding method. The feeding simulation was conducted for 1,000 s, which was sufficient time to finish the feeding. The initial position of individuals on day 1 was randomized using the “Random” function of Unity software. After day 2, the initial position was set according to the final position the day before. We assumed that the fish were well dispersed after the feeding simulation. However, as the spatial interaction between the fish and feed is considered important, we then conducted a simulation in which randomization was performed every day.

The growth simulation was initiated after the feeding simulation. In the growth simulation section, the body mass, $W_{n}^{i}$, and total length, $TL_{n}^{i}$, were calculated using Eqs (14)–(16). After the growth simulation, the feed intake weight of each individual, $S_{n}^{i}$, was 0. The entire simulation was continued until day 90.

The initial body mass and feeding condition were determined based on a previous growth experiment [19]. Here, the initial body mass of all individuals was set to 38.53 g, and the feed intake weight was 1.86% of body mass per day [19]; therefore, the amount of feed distributed per day was 1.86% of the total body mass of 100 individuals. The feed pellet weight *W*_*f*_ was 0.065 g, as mentioned above. The number of pellets given was determined as follows: Amount of feed/*W*_*f*_. The feeding frequency was once per day.

## Results

### Fish behavior in the tank

**Fig 6:**
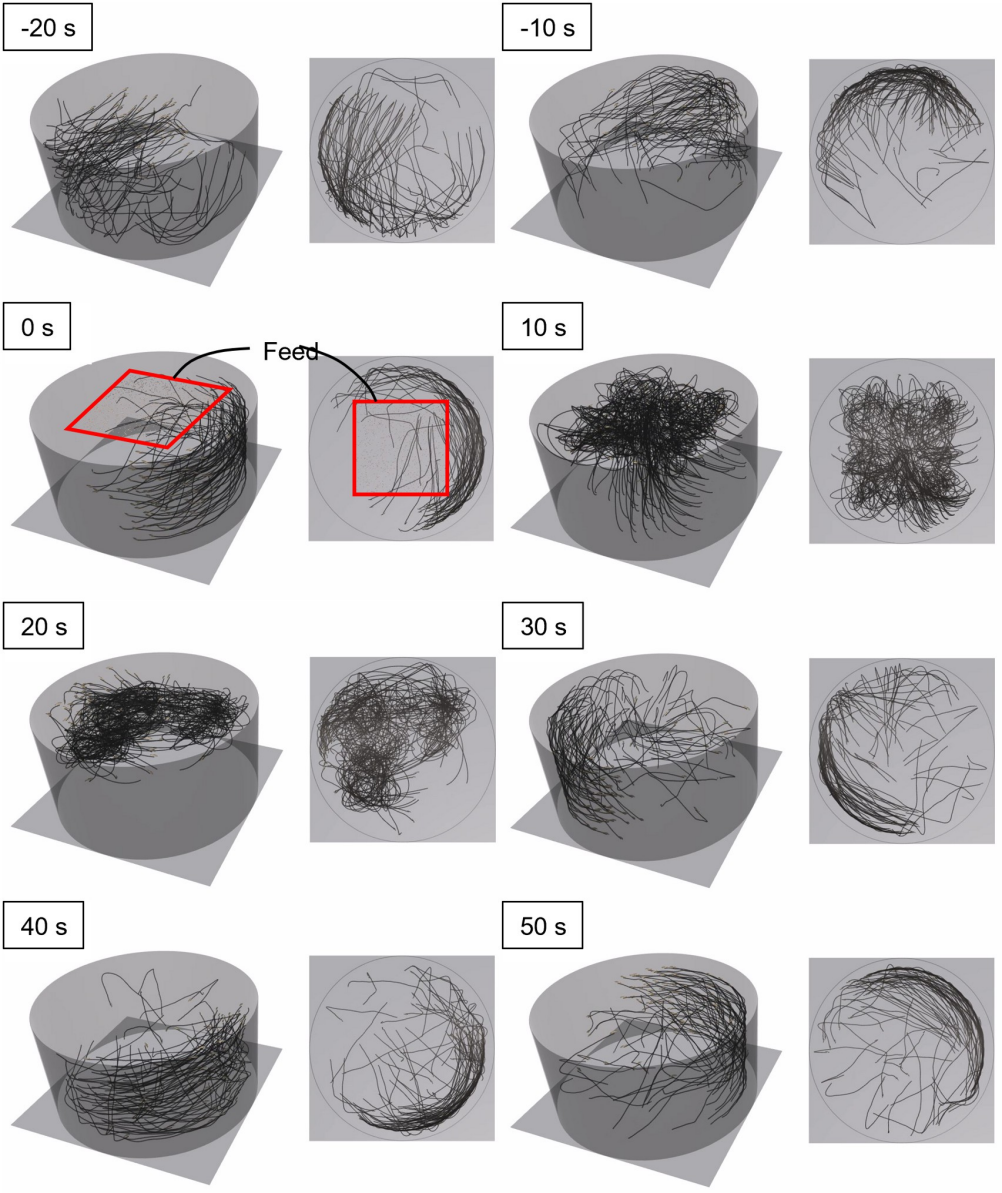
Individual movement paths for the Feeding A group. The elapsed times from the initiation of feeding are indicated.

**Fig 7:**
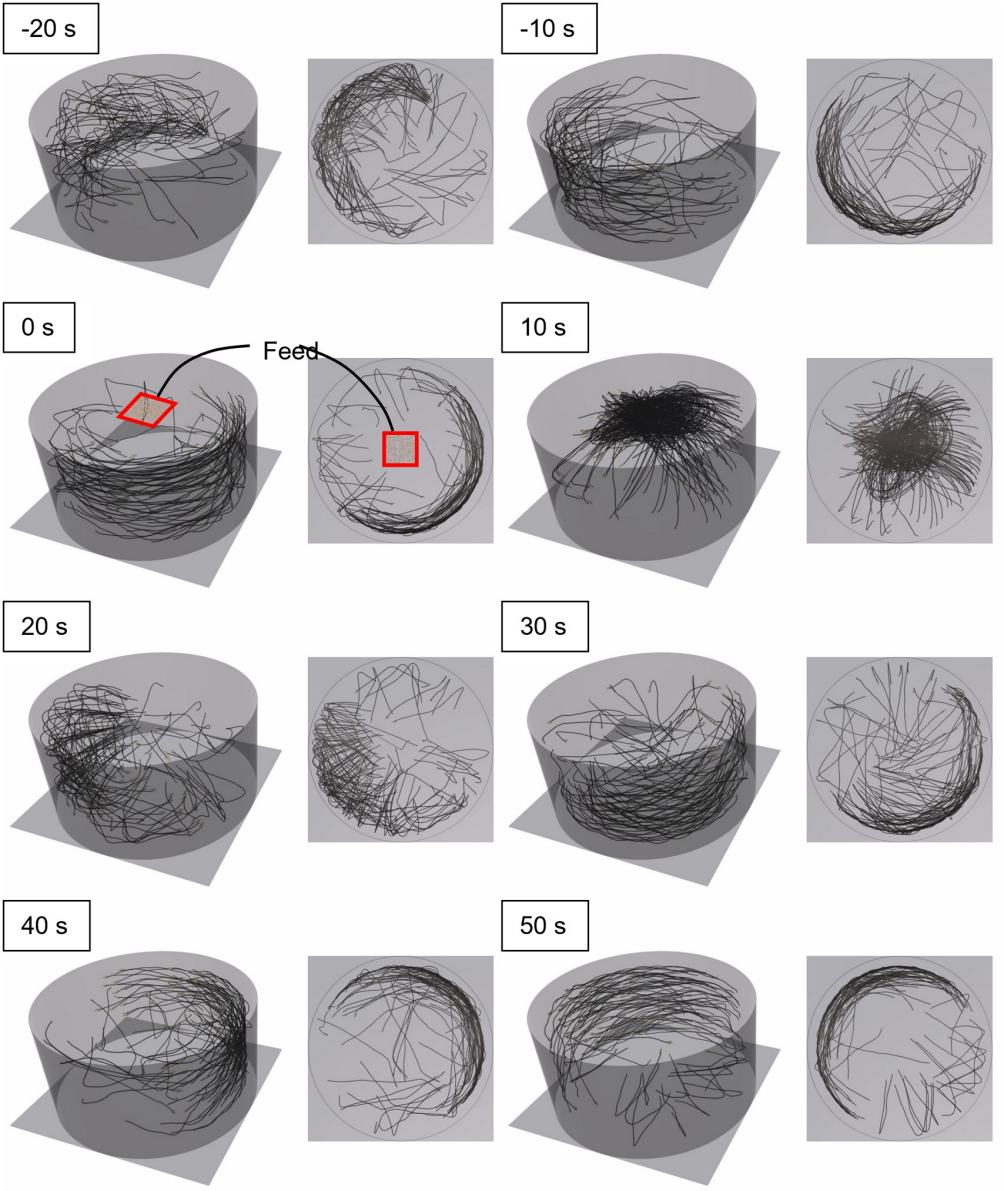
Individual movement paths for the Feeding C group. The elapsed times from the initiation feeding are indicated.

Figs 6 and 7 present the swimming paths of 100 individuals in Feeding A and C groups, respectively.

The individuals formed a school and swam along the wall of the tank (Fig 6). When feeding started (0–10 s), individuals approached, gathered, and ingested the feed. All of the feed was ingested within 30 s. After finishing feeding (40–50 s), the individuals continued standard swimming, and swam near the wall.

The individuals in Feeding C swam along the wall of the tank similar to the Feeding A group (Fig 7). When feeding started (0–10 s), the individuals approached the feed. The individuals gathered in a narrower area compared with that shown in Fig 6. All feed was ingested within 20 s, and the individuals went back to standard swimming.

### Growth of body mass during the simulation

**Fig 8:**
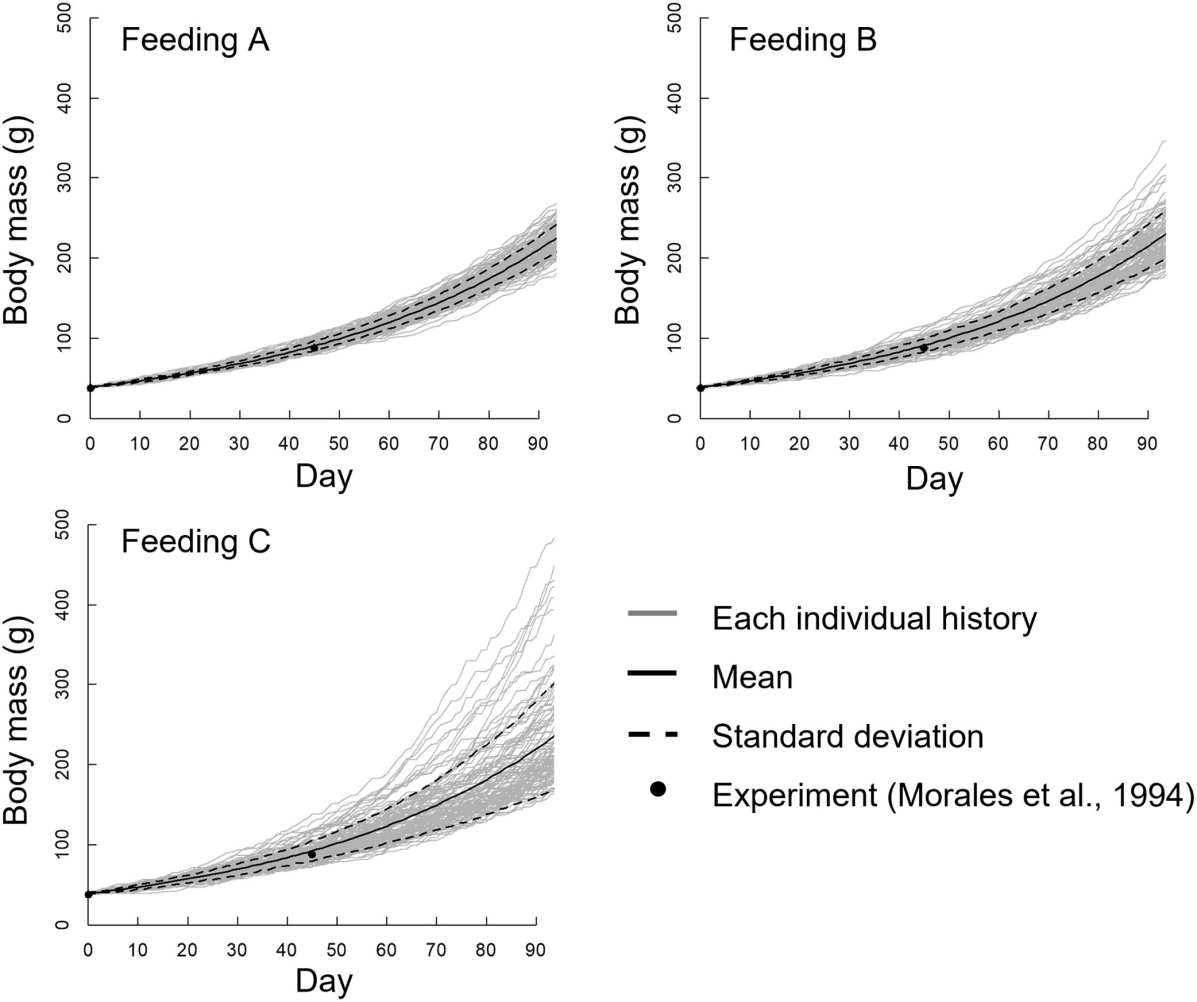
Growth history of the body mass of all 100 individuals using the three feeding methods, without performing randomization every day. The mean and standard deviation of the body mass and previous experimental results [19] are also indicated.

Fig 8 presents the body mass growth histories of all 100 individuals in the three feeding groups; the mean ± standard deviation values and the rearing experimental results of Morales *et al*. [19] are shown.

Body mass increased exponentially in all feeding methods, but the individual difference in body mass increased with the rearing day despite the same initial body mass. In particular, the individual differences in the Feeding C group were very large, and the body masses of several individuals in Feeding C increased dramatically. The mean body mass from the simulation agreed well with the experimental values from Morales *et al*. [19] for all feeding methods, but the standard deviations were different. The standard deviation of the Feeding A group was smaller than that of the other feeding groups, and the standard deviation increased as time elapsed in the Feeding C group.

**Fig 9:**
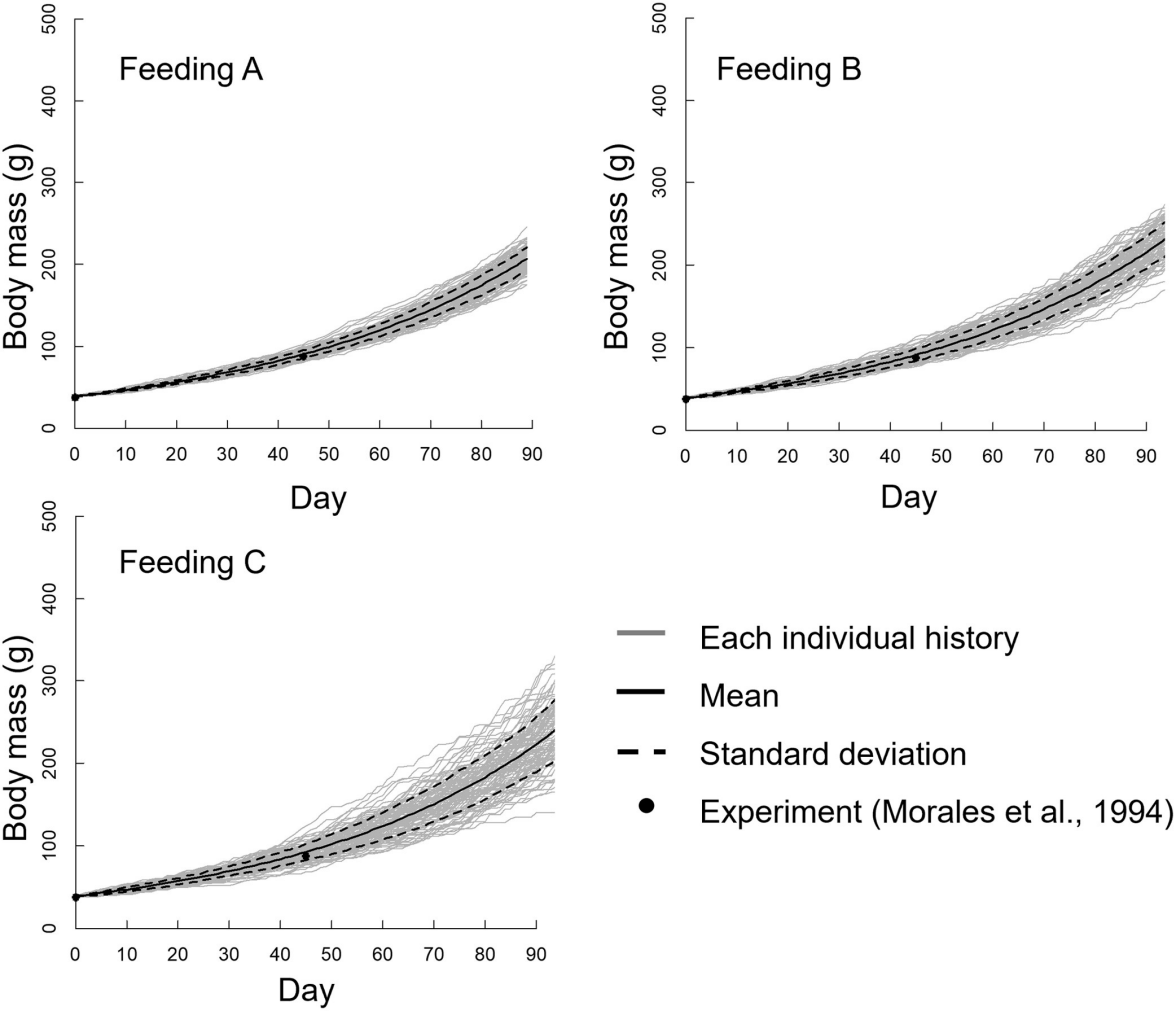
Growth history of the body mass of all 100 individuals using the three feeding methods, with randomization performed every day. The mean and standard deviation of the body mass and previous experimental results [19] are also indicated.

Furthermore, as mentioned in the “Materials and Method”, after the feeding simulation, we conducted a simulation in which randomization was performed every day (Fig 9). The individual difference shown in Fig 9 is smaller compared with that in Fig 8; some remarkably large individuals are shown in Feeding C in Fig 8, but not in Fig 9. However, the tendency for the individual difference to increase with decreasing feeding area was similar between the two figures. Therefore, the following analysis and discussion are based on the results shown in Fig 8.

**Fig 10:**
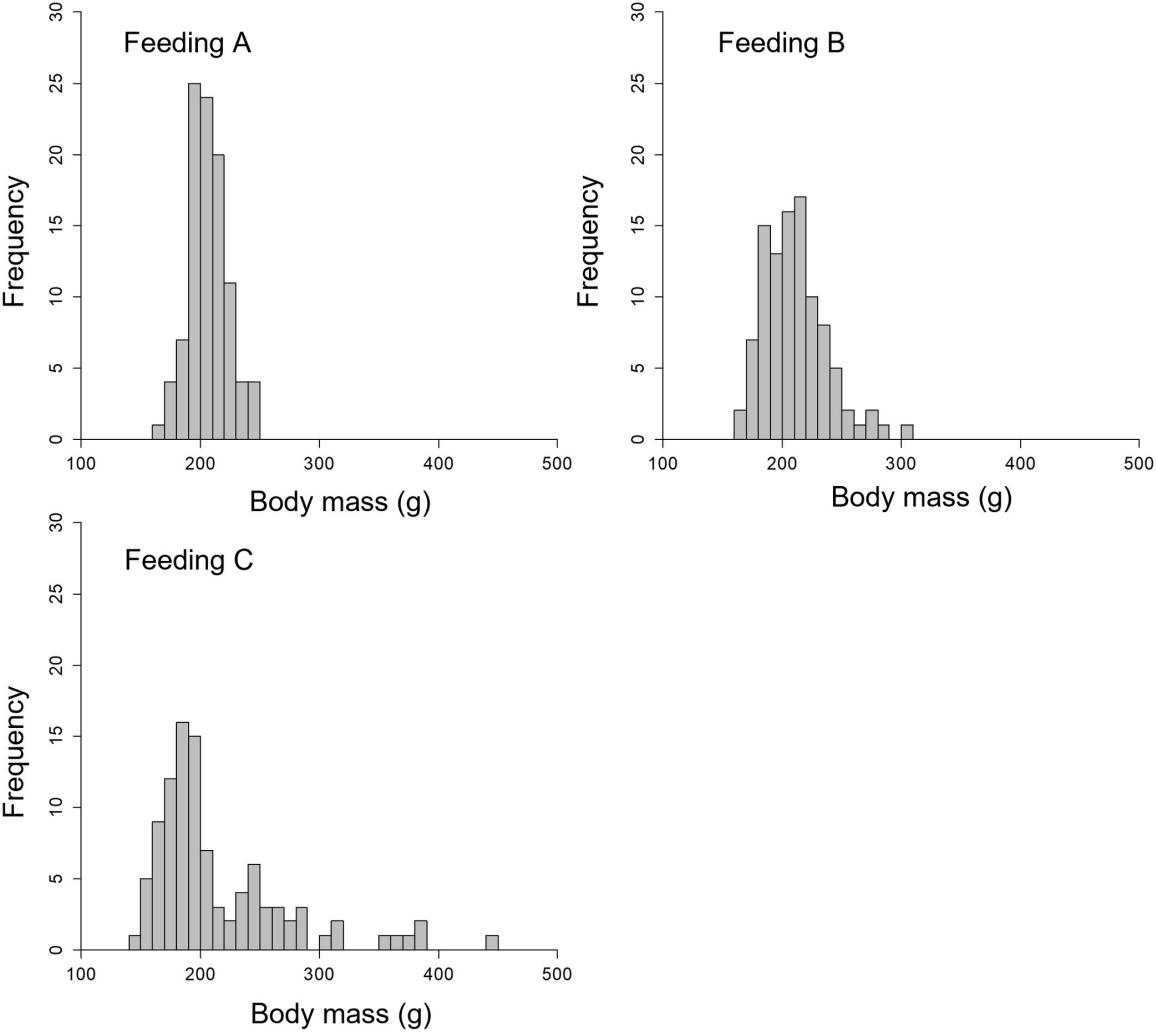
Histogram of the body mass of 100 individuals using the three feeding methods on day 90.

Fig 10 presents the body mass distributions on the final day (day 90) of each feeding method. The mean ± standard deviation values of the final body mass were 210.4 ± 15.7 g for Feeding A, 214.5 ± 27.1 g for Feeding B, and 219.4 ± 59.6 g for Feeding C. The skewness of each distribution was 0.180, 0.914, and 1.700 for Feeding A, B, and C, respectively. The means of body mass were slightly but not significantly different among the three feeding methods (Kruskal-Wallis test, *p* = 0.106). However, a significant difference was detected among the variances of the three distributions (Bartlett test, *p* < 0.001). Consequently, the difference in total body mass among the three feeding methods was not significant regardless of the feeding area. However, the difference appeared in the variance; therefore, the feeding area was affected not by the total mass but by the variability in final growth.

### Individual differences in growth

**Fig 11:**
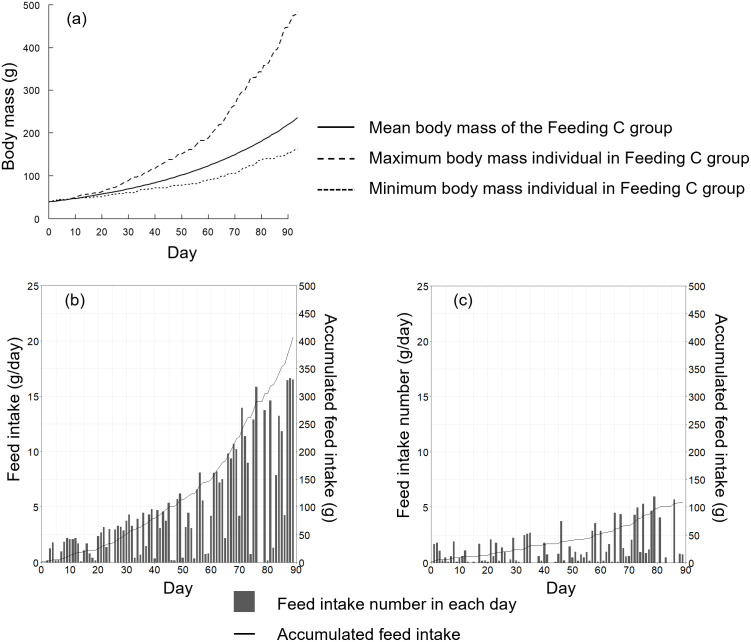
History of the body mass and feeding by the Feeding C group. (a) Growth history of mean weight, maximum weight of an individual, and minimum weight of an individual. (b), (c) Feed intake for each day and accumulated feed intake for the maximum weight individual and the minimum weight individual, respectively.

The individual difference in the body mass differed greatly, especially for Feeding C, the narrow-area feeding method (Fig 10). Fig 11 presents the body mass growth and feeding history of the individuals with the maximum and minimum body masses in Feeding C.

The difference in body mass between the maximum and minimum individuals was small during the early days of the experiment (days 0–20; Fig 11). However, the difference between the two individuals increased gradually. As shown in Fig 11(b) and 11(c), the individual with the maximum body mass ingested feed well; in contrast, the amount of feed ingested by the minimum body mass individual was relatively small. This tendency continued until the final day.

## Discussion

In this study, we developed a simulation model including a feeding behavior and a growth simulation. From the simulation results, the growth variability of the Feeding C group, which was the narrow-area feeding group, was larger than Feeding A, which was the wide-area feeding group (Fig 8). This tendency was also reported by Jørgensen *et al*. [7], who conducted a rearing experiment involving standing and flowing water conditions. The feed clumped in the standing water tank and dispersed in the flowing water tank. As a result, the variability of growth in the standing water (clumped feed) was larger than that in the flowing water (dispersed feed). We demonstrated our hypothesis that the growth difference from feeding the spatial distribution was determined by individual fish behavior under the simulated conditions. The novelty of this study is that the growth of the fish can be explained by individual fish behavior.

The mean body mass in the simulation was compared with a past experimental result, and the body mass growth values agreed well with Morales *et al*. [19]. Similar variability in growth was confirmed in a past study [7]. Thus, we considered that the present simulation results were reliable.

A significant difference was observed between the variance of the body mass of each distribution on day 90 (Fig 10). Additionally, the skewness of narrow-area feeding was larger than that of wide-area feeding. These results suggest that the feeding distribution had an impact on the variability in growth, but not the mean body mass. We visualized the body mass growth and feeding history of the maximum and minimum individuals in Feeding C group; as shown in Fig 11, the individual difference gradually increased along with rearing day. The individual with maximum body mass ingested feed well. In contrast, the individual with minimum body mass did not feed well. Maximum swimming speed in the simulation was proportional to the total length of each individual; therefore, the swimming speed of a large individual was fast. These large individuals accessed and ingested feed more quickly than small individuals, and consequently the larger individuals grew more rapidly. The width of the feeding area was narrow in the Feeding C group, so the few individuals that arrived first to the feeding area dominated feeding activity. This tendency was confirmed by the results shown in Figs 6 and 7, as feeding by the Feeding C group was completed faster than the Feeding A group. As a result, the individual difference in the Feeding C group was larger than the other feeding methods. The process of unequal growth was simulated, which will be useful for aquaculture management.

We now discuss the suitability of the simulation for aquaculture management. Feeding cost is critical when managing an aquaculture farm, so many studies have focused on the optimum feeding amount or methods for fish growth. In the past, an actual rearing test was the only way to determine the optimum feeding amount or method. However, this method is costly and time-consuming. In contrast, the proposed simulation confirmed growth before actual rearing. Therefore, the optimum feeding method and amount can be determined easily on a PC.

Another advantage of the proposed simulation method is that detailed information on the reared individuals can be easily obtained and visualized. Exceptionally large or small individuals always appear during rearing. However, the reasons for this were not clearly understood because it is difficult to obtain an individual growth history. Therefore, we visualized the maximum and minimum body mass growth of individuals and their feeding history, and explored the reasons for growth in detail (Fig 11). Exceptionally large or small individuals have a negative effect on management of an aquaculture farm, because selling price is unstable. A fish farmer can more easily develop a management plan if individual growth history can be predicted. Exceptionally large or small individuals should be excluded for stable management, and predicting the timing is useful for farmers. Therefore, the proposed simulation will be useful for preliminary planning and future management without the need for an empirical method, and will aid to decision making by aquaculture farmers.

One limitation of this study was that some parameters, such as *w*_*n*_, the field of view, and excess feeding ratio, were determined by trial and error. For an accurate simulation, these parameters should be based on experimental results.

The behavioral parameters, *w*_*n*_, and field of view affected the schooling behavior directly. For example, a school will disperse when the value of *w*_1_ (weight of “Separation”) is large and gather when *w*_1_ is small. A recent study proposed a method to measure 3D fish behavior using multi-stereo imaging [25]. In the future, 3D movement paths, including behavior according to feed presence, should be measured to understand interference by each individual of the target species. We believe that our qualitative results are not dependent on these parameters. However, these parameters should be determined from 3D movement paths in laboratory experiments for more accurate simulations.

The growth simulation method has another future task in this study. Here, growth was determined by the *FCE* [19]. Therefore, we estimated the body mass of rainbow trout accurately. However, this method can only be applied to rainbow trout in the same condition as those in the previous experiment. Therefore, the applicability of this simulation is limited. A growth simulation study using the DEB model has been reported [10]. The DEB model was proposed by Kooijman [8] and is based on metabolic theory, including energy and mass budgets for all living organisms at the individual level. The DEB model or another biological model should be used to simulate the rearing conditions without previous experimental rearing information. This should improve the versatility of the simulation.

## Conclusions

In this study, we demonstrated that growth differences according to feeding spatial distribution were determined by individual fish behavior under the simulated conditions. Feeding cost is a critical problem when managing an aquaculture farm. Therefore, many rearing studies have been performed to understand the optimum feeding amount. However, conventional rearing tests are costly and time-consuming. As a result, operation of the actual aquaculture farm relies on the farmer’s experience. Here, a rearing simulation test was easily conducted within a short time using the proposed simulation method, and different methods were attempted on a PC. The results demonstrated that optimal aquaculture operation can be determined without using an empirical method. This simulation is applicable only to rainbow trout under specific conditions. In the future, with the improvements mentioned in the Discussion, we believe that the proposed simulation method can be used as a decision-making tool in aquaculture and will contribute to efficient management of aquaculture farms.

## Supporting information

S1 File Simulated growth and feeding history of Feeding A, without performing randomization every day. (CSV)

S2 File Simulated growth and feeding history of Feeding B, without performing randomization every day. (CSV)

S3 File Simulated growth and feeding history of Feeding C, without performing randomization every day. (CSV)

S4 File Simulated growth and feeding history of Feeding A, with randomization performed every day. (CSV)

S5 File Simulated growth and feeding history of Feeding B, with randomization performed every day. (CSV)

S6 File Simulated growth and feeding history of Feeding C, with randomization performed every day. (CSV)
